# Possible Impact of Vitamin D Status and Supplementation on SARS-CoV-2 Infection Risk and COVID-19 Symptoms in a Cohort of Patients with Inflammatory Bowel Disease

**DOI:** 10.3390/nu15010169

**Published:** 2022-12-29

**Authors:** Amedeo De Nicolò, Jessica Cusato, Cristina Bezzio, Simone Saibeni, Marta Vernero, Michela Disabato, Gian Paolo Caviglia, Alice Ianniello, Alessandra Manca, Antonio D’Avolio, Davide Giuseppe Ribaldone

**Affiliations:** 1 Laboratory of Clinical Pharmacology and Pharmacogenetics, Department of Medical Sciences, University of Turin, 10149 Turin, Italy; 2 Gastroenterology Unit, Rho Hospital, ASST Rhodense, 20017 Milan, Italy; 3 Gastroenterology Unit, Department of Medical Sciences, University of Turin, 10126 Turin, Italy

**Keywords:** vitamin D, cholecalciferol, IBD, Crohn’s disease, ulcerative colitis, monoclonal antibodies, SARS-CoV-2, COVID-19

## Abstract

The coronavirus disease (COVID-19) pandemic represents a global health challenge, particularly considering concomitant diseases. Patients with inflammatory bowel diseases (IBD) can be considered a population at risk. On the other hand, the risk of developing IBD and COVID-19 have both been described as modulated by vitamin D (VD) levels. In this work, a cohort of 106 adult patients affected by IBD was prospectively enrolled, during the second wave of the pandemic in Italy. In these patients, VD plasma levels, demographic, and clinical characteristics were tested for a correlation/an association with the risk of infection with SARS-CoV-2 in the study period (anti-spike IgG positivity) and the severity of COVID-19 symptoms. By multivariate logistic regression analysis, VD supplementation (Odds Ratio; OR 0.116, *p* = 0.002), therapy with monoclonal antibodies (OR 0.227, *p* = 0.007), and the use of mesalazine (OR 2.968, *p* = 0.046) were found to be independent predictors of SARS-CoV-2 positivity. Moreover, hypertension was associated with severe disease (*p* = 0.019), while a VD level higher than 30 ng/mL (*p* = 0.031, OR 0.078) was associated with asymptomatic infection. No interplay between IBD activity and COVID-19 risk of infection or symptoms was observed. These results confirm the importance of VD levels in defining the risk of COVID-19 and give encouraging data about the safety of maintaining immunomodulatory treatments for IBD during the COVID-19 pandemic.

## 1. Introduction

Inflammatory bowel diseases (IBD) are a heterogeneous group of diseases including Crohn’s disease (CD), ulcerative colitis (UC), and unclassified IBD (IBD-U). They are characterized by a chronic relapsing course and multifactorial etiopathogenesis [1]. IBDs are considered an emerging global disease, and their incidence is increasing in developing countries. It is estimated that more than three million people currently suffer from IBD in Europe, and more than five million worldwide [1,2]. This trend is influenced by the rapid industrialization and urbanization of vast Eastern areas; in fact, incidences IBD of 1.37 × 10^5^ in Asia and 3.4 × 10^5^ in China are observed (an increase compared to the traditional incidence of 0.60–3.44 × 10^5^) [1,2]. Since December 2019, the pandemic of SARS-CoV-2 (a betacoronavirus related to SARS-CoV) [3], causing coronavirus-19 disease (COVID-19), has become a global health challenge, even complicating the management of other diseases, such as IBD. With COVID-19, the risk factors which were shown to be associated with the worst prognosis are age > 65, cigarette-smoking, diabetes, hypertension, cardiovascular disease, chronic lung disease, malignancy, and immunosuppression [4]. The clinical picture occurs as SARS-CoV2 binds to the ACE2 receptor, which is particularly expressed in the lungs. Following an incubation period of about 4 to 14 days, most people develop symptoms ranging from mild to very severe, and sometimes even fulminant, illness. The most common manifestations are cough (46–82%), fever (77–98%), fatigue, anorexia, and myalgia. Gastrointestinal symptoms such as nausea, diarrhea, and abdominal pain may precede respiratory symptoms in approximately 10% of patients. About 30% of patients are asymptomatic [5].

Cases of severe COVID-19 were seen more frequently in patients with gastrointestinal symptoms (23%) than in patients who did not (8%). This may be due to increased mucosal cytokine production [6]. In addition, the presence of diarrhea is thought to be linked to direct infection of the gastrointestinal cells [7]. Esophageal, gastric, duodenal, and rectal epithelial cells have been shown to express the ACE2 receptor; in fact, plasma cells and lymphocytes indicating the activation of immune cells of the mucosa have been observed in the lamina propria of these organs [8]. Vitamin D (VD) is a fat-soluble vitamin with a pleiotropic effect on many tissues and organs, and is today considered a pro-hormone [9]. Among the most important biological roles of VD are controlling bone rearrangement, having an effect on both osteoblasts and osteoclasts, and absorbing calcium and phosphorus intestinally, facilitating their homeostasis [9,10]. Nevertheless, many additional roles have been described for VD, from drug metabolism to the control of the immune response [1,9,11,12,13]. In particular, VD can affect the onset and disease activity of IBD by enhancing the mucosal barrier, increasing the differentiation of T cells into Tregs and by affecting gut microbiota [1,14]. Low levels of 25(OH)-VD (the main circulating form) in plasma can increase the likelihood of developing disorders such as cardiovascular and autoimmune diseases, diabetes, and infectious diseases [13,14,15,16,17,18,19]. Some authors have hypothesized that VD may down-regulate ACE-2 receptors and antagonize their binding to the spike protein of SARS-CoV-2, and therefore may have protective effects against COVID-19 [20,21]. VD deficiency has been linked to susceptibility to SARS-CoV-2 infection and COVID-19 severity [22,23,24,25,26,27]. Nevertheless, different studies have found conflicting results on this association, and this may be partly due to the heterogeneity of the study designs and of the studied populations, and the lack of correction for comorbidities [28,29]. In fact, the interplay between COVID-19, the underlying disease, and the relative treatment could affect the role of VD levels and supplementation on the infectious-risk-and-disease severity. The aim of our study was to evaluate the relationship between serum VD levels and supplementation, and SARS-CoV2 infection, COVID-19 symptoms, IgG positivity, and titers in a cohort of patients with IBD, considering possible interplays with the clinical characteristics of IBD.

## 2. Materials and Methods

### 2.1. Patients’ Enrolment, Inclusion Criteria, and Data Collection

We conducted a prospective, observational, multi-center experimental case-control study in which we randomly and consecutively recruited patients with IBD. The study was approved by the ethics committee on 12 November 2020 (protocol number 0109499).

Inclusion criteria: a confirmed diagnosis of IBD according to the guidelines of the European Crohn’s and Colitis Organization (ECCO) [30]; age over 18 years old; willingness to participate in the study and signing of informed consent.

Exclusion criteria: execution of the anti-SARS-CoV-2 vaccine, due to the inability to discriminate between the increase in IgG because of the vaccine, and the increase due to SARS-CoV-2 infection.

Primary outcome was the evaluation of the correlation between vitamin D levels and SARS-CoV-2 infection (anti-SARS-CoV-2 IgG positivity) and COVID-19 symptoms. Secondary outcomes were as follows: correlation between IBD-related clinical variables and therapeutic options and previous COVID-19 SARS-CoV-2 infection (anti-SARS-CoV-2 IgG positivity) and/or COVID-19-related symptoms; correlation between patients’ characteristics and serological evidence of SARS-CoV-2 infection (anti-SARS-CoV-2 IgG positive) in the previous months, and symptomatic COVID-19.

We created an Excel database by entering the following parameters:

- General: age, sex, smoking, comorbidities (diabetes and obesity);
- Clinical history: type of IBD (CD, UC, IBD-U), year of diagnosis of IBD, topographical location, drug therapy taken (no therapy, topical or systemic steroid therapy, biological drug), comorbidity;
- IBD clinical activity: remission, mild, moderate, and severe. Evaluated on the basis of two different scores, the Mayo Clinic Partial in patients with UC and the Harvey– Bradshaw Index in patients with CD;
- Vitamin D data: dose of VD taken, level of 25-hydroxy (OH) VD in serum;
- COVID-19 data: serological evidence of previous SARS-CoV-2 infection (positive or negative SARS-CoV-2 IgG and anti-SARS-CoV-2 IgG dose), COVID-19 symptoms (fever, cough, dyspnea, diarrhea, vomiting, headache, anosmia, ageusia, pneumonia, hospitalization, ventilation, sequelae), type of therapy taken for COVID-19, any changes in therapy for IBD during infection. Patients with a doubtful anti-SARS-CoV-2 IgG level, having a positive history of virus infection, were considered positive. Severity on COVID-19 was divided into 3 groups: asymptomatic COVID-19 (absence of clinically appreciable symptoms), mild COVID-19 (at least 1 among cough, rhinitis, fever, anosmia/ageusia), and severe COVID-19 (patients with pneumonia/hospitalization).

Patients’ enrolment and blood sampling were performed from January to April 2021 at “Città della Salute e della Scienza” Hospital, Turin, and at Rho Hospital, Milan. The visit and the blood collection were carried out after the verification required by law of the absence of symptoms compatible with COVID-19 in the previous 2 weeks. LIAISON SARS-CoV-2 S1/S2 IgG serological test was used to evaluate the positivity of IgG antibodies against SARS-CoV-2 at the Department of Microbiology of the “Città della Salute e della Scienza” Hospital, Turin. Patients with negative SARS-CoV-2 tests were recruited as controls. The quantification of 25-OH VD (25(OH)-VD) levels in plasma was performed by ultra-high-performance liquid chromatography coupled with tandem mass spectrometry (UHPLC-MS/MS) at the Laboratory of Pharmacology and Clinical Pharmacogenetics, “Amedeo di Savoia” Hospital, Turin. The patients were then classified in three classes of VD status, according to the guidelines of the US Institute of Medicine and the US Endocrine Society [31,32]: VD-insufficient (concentrations < 20 ng/mL), intermediate (between 20 and 30 ng/mL), and optimal (>30 ng/mL).

### 2.2. Statistical Methods

Continuous variables were tested for normality; the normality of the data was checked with the D’Agostino–Pearson test. Continuous variables normally distributed were reported as mean ± standard deviation (SD). Continuous variables not normally distributed were reported as median and interquartile range (IQR).

The categorical variables were reported as number and percentage. The Mann–Whitney test was used for evaluating differences in non-normally distributed continuous variables between categorical groups, while Chi-square test was used for testing associations between categorical variables.

Variables which resulted in a significant association with positivity and/or COVID-19 severity by Chi-square test were then tested for their predictivity (Odds Ratio, OR) by univariate and multivariate logistic regression analysis. Receiver Operating Characteristic (ROC) curve was used to identify putative cutoff values. The main endpoint of this study was testing the association/predictivity of patient-related characteristics with recent history of COVID-19 infection, tested by serology, and with the severity of COVID-19 symptoms. The independent variables tested for these associations were as follows: age, sex, smoking, type of IBD, IBD activity, hypertension, weight, VD status, and supplementation. Since the time of writing the study protocol (7/2020), there was no study in the literature that evaluated the association between VD levels and the incidence of anti-SARS-CoV-2 IgG positivity; thus, it was not possible to perform an accurate power calculation to determine the optimal sample size a priori.

All statistical analyses were performed using SPSS software 18.0 (IBM, Armonk, NY, USA), and a *p* value < 0.05 was considered statistically significant.

## 3. Results

### 3.1. Patient Characteristics

In this prospective observational study, 106 patients were enrolled, 69 from the “Città della Salute e della Scienza” Hospital, Turin, and 37 patients from the Rho Hospital, Milan.

Among these patients, 68 patients had Crohn’s disease (CD), 39 ulcerative colitis (UC), and 4 IBD-U. The vast majority of these patients were in the remission state (63, 59.4%) or had mild symptoms (32, 30.2%), while only a minor fraction had moderate (8, 7.5%) or severe (3, 2.8%) disease activity. Upon enrolment, 24 had a positive anamnesis of SARS-CoV2 positivity (molecular testing) in the previous 3–4 months and 30 resulted in a positive serological test. Considering the 24 patients with a known anamnesis of COVID-19 (positivity to SARS-CoV-2 PCR testing), five were asymptomatic, 15 had mild symptoms, and four had severe symptoms; the possible timing and symptoms of SARS-CoV2 and COVID-19 were not assessable for the remaining six patients who tested positive only in the serological testing (albeit surely not severe). A summary of the baseline patients’ characteristics is provided in Table 1.

### 3.2. General Patients’ Characteristics and COVID-19

Among the patients, female patients appeared more frequently positive in the anti-SARS-CoV-2 serological test compared to male patients, with borderline significance (*p* = 0.053), but no significant differences appeared in terms of the severity of the COVID-19 symptoms. Furthermore, age did not show any significant association with positivity or severity (*p* = 0.895 and 0.237), probably due to the small sample size and the relatively young age of these patients. Similarly, no significant differences were observed with regard to the smoking status and COVID-19 positivity or symptom severity (*p* = 0.735 and 0.814, respectively). Among non-IBD comorbidities, the only significant association was found between severe COVID-19 and hypertension (three out of four severe COVID-19 cases had hypertension, *p* = 0.019).

### 3.3. IBD Features, Treatment, and COVID-19

**Figure 1:**
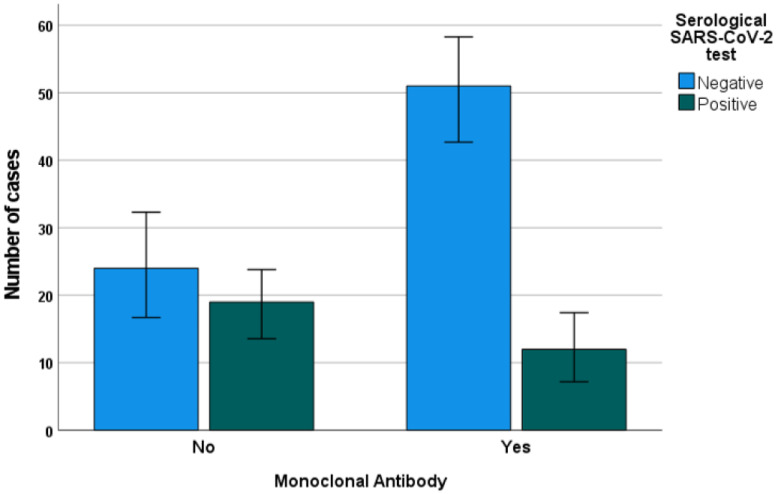
Frequency of serological positivity for SARS-CoV-2 antibodies among IBD patients receiving or not receiving therapy with monoclonal antibodies. Whiskers represent 95% error bars.

No significant associations were found between the type of IBD (CD vs. UC vs. IBD-U) and COVID-19 severity (*p* = 0.446). Similarly, no significant association was found between the IBD activity score and the COVID-19 severity (*p* = 0.261) or positivity in the serological test (*p* = 0.299). Conversely, positivity in the SARS-CoV-2 serological tests was differentially distributed between the different types of IBD (*p* = 0.050), with significantly more positive patients with UC or IBD-U, compared to CD. The use of corticosteroids was not significantly associated with positivity in the serological tests or with the severity of COVID-19 symptoms (*p* = 0.897 and 0.476, respectively). Strikingly, significant positive and negative associations with positivity in the serological tests, respectively, were found between the use of mesalazine (*p* = 0.019) and monoclonal antibodies (Figure 1, *p* = 0.005, *p* = 0.074 for anti-TNF drugs, not significant for other biologics alone), but not with the severity of COVID-19 symptoms (*p* = 0.153 and 0.671). The administration of mesalazine was significantly more common among UC and IBD-U patients, compared with CD (*p* = 0.005), possibly explaining the mutual association with positivity in the serological SARS-CoV2 test, while no association was found with monoclonal antibodies (*p* = 0.280).

### 3.4. Vitamin D Concentration, Supplementation, IBD, and COVID-19

**Figure 2:**
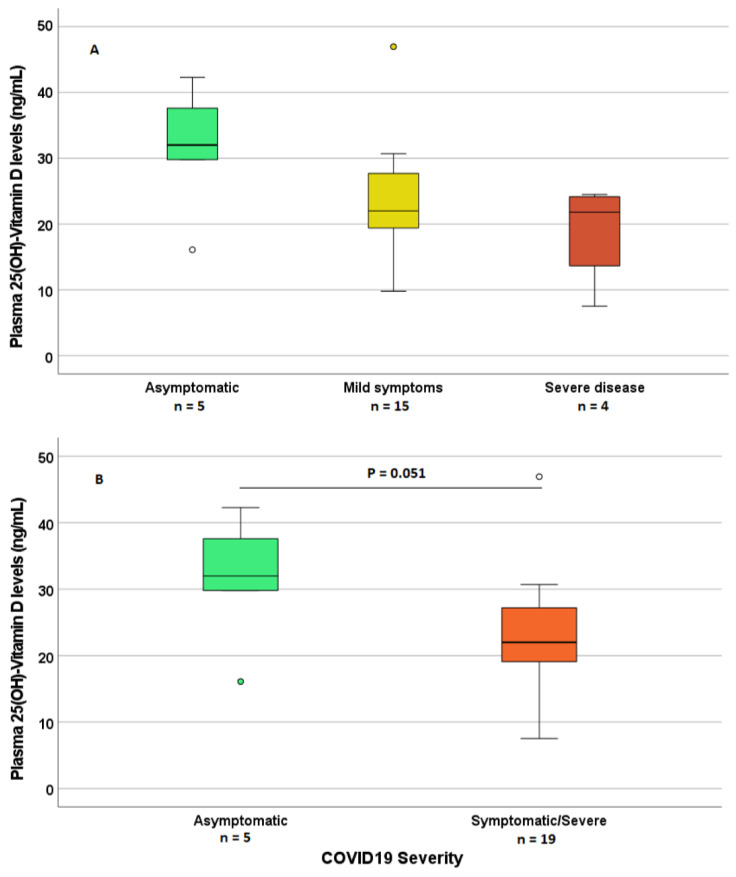
Differences in total 25(OH)-VD plasma concentrations according to COVID-19 symptoms ((**A**) comparison among Asymptomatic, Mild symptoms, Severe disease; (**B**) comparison between Asymptomatic versus Symptomatic + Severe). Circles are outlier samples.

Considering the 25(OH)-VD concentrations in plasma, no significant association was found with positivity in SARS-CoV2 testing. Conversely, 25(OH)-VD concentrations were higher in asymptomatic patients (borderline significance, *p* = 0.051), compared with the ones with mild or severe symptoms (Figure 2). 

Using an ROC curve, a cutoff 25(OH)-VD concentration of 30 ng/mL was observed to be associated with a higher probability of asymptomatic infection (*p* = 0.049, sensitivity 66.7%, specificity 89.5%). No differences in 25(OH)-VD concentrations were found between different types of IBD (*p* = 0.290) or IBD activity score, but it was observed that VD supplementation was more frequent in patients with CD, rather than those with UC or IBD-U. In turn, VD supplementation (as well as CD) was significantly less common among patients who tested positive in the SARS-CoV2 serological test (*p* = 0.047). VD supplementation resulted in significantly higher 25(OH)-VD concentrations (*p* = 0.012).

**Figure 3:**
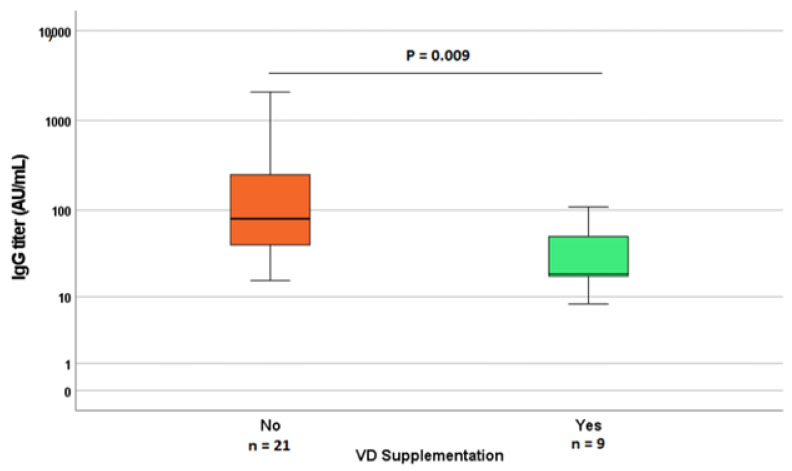
Difference in Anti-SARS-CoV-2 S1/S2 IgG titers between patients taking or not taking VD supplementation.

Deepening this issue, among patients with positive serological tests, the anti-SARS-CoV2 IgG titers showed a lower result in the group of patients who had VD supplementation (Figure 3), compared with other patients. Similarly, the IgG titers showed a higher result in patients with the worst symptoms (*p* = 0.040 for the comparison between severe cases vs. others; *p* = 0.084 for the comparison between all COVID-19 severity classes) compared with asymptomatic or mild COVID-19.

### 3.5. Predictors of COVID-19 Positivity/Severity

Based on the observed results, we refined the analysis by using a univariate and multivariate binary logistic regression analysis for the prediction of SARS-CoV-2 serological positivity. The results, with significance values and the observed odds ratios (OR, with 95% confidence intervals), are reported in Table 2. 

The only significant mutually independent predictors which were retained in the final model were the use of mesalazine (*p* = 0.046; OR 2.968 CI95% 1.021–8.623]), monoclonal antibodies (*p* = 0.007; OR 0.227 CI95% 0.078–0.662), and VD supplementation (*p* = 0.002; OR 0.166 CI95% 0.053–0.517). On the other hand, with regard to COVID-19 symptoms, the only significant negative predictor for symptomatic disease was having 25(OH)-VD concentrations > 30 ng/mL (*p* = 0.031; OR 0.078 CI95% 0.008–0.792).

## 4. Discussion

Vitamin D plays an important role within the immune system as, by interacting with the vitamin D receptor (VDR) within immune cells, it promotes the transcription of antimicrobial peptides such as beta defensins and cathelicidins. Cathelecidins are responsible for both the destruction of bacterial cell membranes and enveloped viruses, i.e., viruses protected by a rich layer of lipids, such as SARS-CoV-2. On the other hand, beta defensins promote the chemotaxis of inflammatory cells due to an increase in capillary permeability [21,28,33]. Vitamin D is capable of promoting the activity of regulatory T cells (Tregs) and a partial transition from the Th1 to Th2 response: these changes can be capable of reducing the levels of pro-inflammatory cytokines (IL-1, IL-6, IL-12, IL-17, and TNF-α), and increasing the levels of anti-inflammatory cytokines (IL-10) [16,34]. In particular, IL-6 and TNF-α are the two key cytokines involved in the development of the cytokine storm underlying acute respiratory distress syndrome (ARDS), which can be down-regulated by VD [1,35,36]. It has also been hypothesized that VD may down-regulate ACE-2 receptors and antagonize its binding to the spike protein of SARS-CoV-2 [20,21]. All these factors, taken together, can explain the lower frequency and severity of COVID-19 symptoms in the patients with higher 25(OH)-VD concentrations, which was observed in this study.

Particularly, it is interesting to note that, by ROC curve analysis, the cutoff of 30 ng/mL was found to be protective against COVID-19 symptoms, which is the same amount proposed in a few previous works regarding COVID-19 and in the guidelines of the US Endocrine Society (for extra-bone effects) [10,24,32,37]. Vitamin D deficiency is also a risk factor involved in the pathogenesis of IBD [1]. This derives from the dual action of vitamin D, which, on the one hand, promotes an anti-inflammatory response and, on the other, guarantees the barrier function of the intestinal mucosa through the expression of proteins such as claudin-1 and occludin responsible for the junctions between intestinal epithelial cells [1,38]. Nevertheless, in this study, no significant differences were found in the VD levels between the types of IBD or disease activity, despite a significantly higher prevalence of VD supplementation present among patients with CD. Among the 106 enrolled patients, 30 (28%) tested positive in the SARS-CoV-2 serological test. This percentage does not reflect the seroprevalence levels found in the general population in that same period (equal to 2.5%, ISTAT [39]), but this discrepancy can be explained by the prevalent enrolment of patients who reported a positive history of COVID-19, along with other patients who underwent their regular follow-up for IBD. In our study, we evaluated the presence or absence of comorbidities, paying more attention to arterial hypertension and obesity: within our study sample, hypertension was present in six out of 106 patients (5.7%) and obesity in only one of the patients analyzed (0.9%). This low prevalence of hypertension in our sample compared with the general population (from 27% under 60 years old to 74% above 80 years old) can be explained by the relatively low observed median age of 45 years old (IQR 35–56), which is expected from the literature-derived data on the age of onset of IBD (15–25 years old for CD; 25–35 for UC) [40]. Moreover, considering that IBD causes a higher risk of malnutrition due to reduced food intake, malabsorption, and protein loss, the risk of obesity (and hypertension) is reduced further [41]. Nevertheless, it is important to note that, in the group of patients with a history of COVID-19, severe illness was significantly associated with hypertension (three out of four patients, *p* = 0.019), but these data should be taken with caution due to the low number of patients with severe COVID-19 in our study.

Considering the levels of IgG, we observed that patients with milder COVID-19 symptoms and with VD supplementation had significantly lower IgG titers, in accordance with previous studies, which denoted a positive association between the humoral response and the severity of COVID-19 symptoms and/or patients’ age [42,43,44]. In contrast with other cohorts, in this study, we did not observe any significant difference in age between symptomatic or asymptomatic patients; again, this discrepancy could be due to the younger age in our cohort, while the higher risk of a worse prognosis is associated with an age above 65 years. Similarly, smoking status was associated with neither COVID-19 positivity nor its symptoms’ severity, probably due to the low percentage of patients who smoke. In our cohort, a higher frequency of symptoms was found, but they tend to be mild; again, this can be due to selection bias caused by the enrolment of patients with a known history of COVID-19 (mostly symptomatic in the first two waves of the COVID-19 pandemic). In this study, no significant differences in VD levels were found between patients who tested positive or negative in SARS-CoV-2 serological tests, in contrast with previous studies [23]: this can be due to the possible effect of behavioral differences between patients in the period of observation. In fact, patients who observed a stricter lock-down could have been more protected from SARS-CoV-2 infection, while still having lower VD levels (considering lower sunlight exposure). This hypothesis seems to be confirmed by the fact that, instead, oral VD supplementation appeared to be negatively associated with positivity in the serological test (*p* = 0.047). Moreover, it is important to note that the Ab titers were lower in patients with VD supplementation and higher VD levels, possibly leading to more false negative results in the long term. These challenges and sources of bias in studying the link between VD concentrations and SARS-CoV-2 epidemiology can explain the widely discordant evidence between studies [28,29]. In fact, while the results from observational cross-sectional studies generally indicate that VD insufficiency is strongly associated with SARS-CoV-2 infection and severity [22,23,28,29,45,46], evidence from interventional RCTs on VD supplementation in COVID-19 are more discordant, both in terms of prophylactic [29,47] and therapeutic effectiveness [48,49,50,51]. In our cohort, the absence of extremely impactful differences in VD status between positive and negative patients and COVID-19 severity classes can also be due to the low frequency of extremely low VD levels among our patients, who in most cases already take VD supplementation (40.6%).

On the other hand, in patients with symptomatic COVID-19, the levels of VD tended to be lower than in patients with asymptomatic COVID-19: 22.9 (17.8–27.7) vs. 32.2 ng/mL (IQR 30.0–40.0). This difference resulted in a borderline significance (*p* = 0.049). This trend is in line with studies in which, for example, fever was significantly higher in patients with VD-deficient levels than in those with VD levels within the normal levels [52]. 

The presence of COVID-related symptoms was not associated with severe IBD activity (*p* = 0.261). Usually, IBD patients have an increased risk of infection, especially when on steroid, immunosuppressant, or biologic therapy. In our study, we did not observe an increased risk in patients on immunosuppressive therapy of developing COVID-19-related symptoms. Conversely, we found a negative association between the use of monoclonal antibodies (particularly anti-TNF drugs) with positivity in the serological test, suggesting a possible protective role against infection: this could be due to a reduction of ACE-2 expression or behavioral differences in those patients who underwent therapy with biologics (e.g., stricter observance of lock-down) [53]. Regarding the hypothesis of a possible modulation of ACE-2 expression by TNF-α, a recent study evidenced a synergistic effect of TNF-α and IFN-γ in increasing ACE-2 expression in human thyroid cells [54]; nevertheless, it is not possible to directly translate these data on epithelial lung cells in patients with IBD, who could have a higher TNF-α background signaling. From a clinical point of view, in line with our findings, the studies by Bezzio et al. showed that complications and lethality in IBD patients reflect the natural history of the infection and are not related to monoclonal antibodies or other immunosuppressants [55,56]. In particular, in the literature, it has been seen that anti-TNF drugs, widely used in the therapy of IBD, have a potential protective effect against COVID-19 symptoms due to a higher production of Tregs and, consequently, of anti-inflammatory cytokines. Furthermore, the use of vedolizumab and ustekinumab is not related to a marked increase in viral infections in patients with IBD [57]. Thus, there is no evidence to suggest that patients with IBD should discontinue disease-specific drug therapy. In fact, the International Organization for the Study of Inflammatory Bowel Diseases (IOIBD) suggests continuing with maintenance therapy to avoid severe IBD flare-ups [58]. 

Our study has some limitations. Among these, the statistical power was limited by the small sample size, so further studies with larger case series will be needed to compare these results. Moreover, the observational and cross-sectional nature of this study does not allow us to determine any cause–effect relationship between VD status and COVID-19. Finally, since a very limited number of patients had a severe COVID-19 course (only four were hospitalized, and only one underwent active ventilatory support), no significant association was found between VD levels and severe disease; a signification association was only found between VD levels and the absence or presence of COVID-19 symptoms. Furthermore, most of the patients we considered were already taking VD supplementation, reducing the number of patients with extremely low VD levels.

## 5. Conclusions

In conclusion, our study confirmed that VD levels were associated with a protective effect against COVID-19 symptoms even in IBD patients, probably due to VD’s intrinsic anti-inflammatory effect. Moreover, VD supplementation was shown to be negatively associated with positivity in SARS-CoV-2 serological testing. On the other hand, our study also reassures us about the safety of immunomodulators and, particularly, biological drugs in the treatment of intestinal pathology, since they did not increase the risk of developing COVID-19-related complications, but, on the contrary, prevent IBD exacerbations. Interestingly, VD supplementation and IBD treatment with biological drugs and/or mesalazine were shown in our results to be independent predictors of the risk of testing positive for SARS-CoV-2 infection, suggesting that the predictive value of VD in COVID-19 is not affected by the underlying treatment of IBD.

## Figures and Tables

**Table 1:** Summary of patients’ characteristics on the day of enrolment. y.o. = years old; IQR = interquartile range; IBD = inflammatory bowel disease; CD = Crohn’s disease; UC = ulcerative colitis; IBD-U = unclassified IBD; TNF = tumor necrosis factor; IL = interleukin.

Total Number	106
Gender [Male–Female]; n (%)	63/43 (59.4%/40.6%)
Median Age (y.o.; IQR)	45 (38–56)
Median years from IBD diagnosis (IQR)	12.0 (5.8–20.0)
Smoke [yes/ex/no] (%)	17/25/64 (16.0%/23.6%/60.4%)
Type of IBD [CD–UC–IBD-U]; n (%)	63/39/4 (59.4/36.8/3.8)
Use of Mesalazine; n (%)	60 (56.6%)
Use of corticosteroids; n (%)	13 (12.3%)
Use of Monoclonal Antibodies [yes/no]; n (%)	63/43 (59.4%/40.6%) Anti-TNF: 30 Vedolizumab: 20 Ustekinumab: 7 Anti-IL-23: 6
Vitamin D supplementation (yes/no)	43/63 (40.6%/59.4%)
Median Vitamin D levels (ng/mL; IQR)	21.9 (14.8–28.7)
Vitamin D status (<20 ng/mL; 20–30 ng/mL; >30 ng/mL); n (%)	45/38 /23 (42.5%/35.8%/21.7%)
COVID-19 Anamnesis (yes/no); n (%)	24/82 (19.8–80.2)
COVID-19 Severity [No sympt./Mild/Severe]; n	5/15/4
Serological Anti-SARS-CoV2 Ag [Positive/Negative] (%)	30/76 (28.3%/71.7%)
Anti-SARS-CoV2 titer (AU/mL)	4.81 (3.80–18.05)

**Table 2:** Summary of the estimated Odds Ratios determined by univariate and multivariate logistic regression analysis for the prediction of positivity in the SARS-CoV-2 serological test.

Univariate Logistic Regression Analysis		
Predictor	*p* Value	Odds Ratio (Conf. Interval 95%)
Vitamin D supplementation (yes = 1; no = 0)	0.050	0.395 (0.156–1.000)
Use of Monoclonal Antibodies (yes = 1; no = 0)	0.012	0.327 (0.136–0.783)
Use of Mesalazine (yes = 1; no = 0)	0.032	2.750 (1.090–6.940)
Crohn’s Disease (yes = 1; no = 0)	0.036	0.398 (0.168–0.943)
Sex (Male = 1; Female = 0)	0.036	0.398 (0.168–0.943)
**Multivariate Logistic Regression analysis**		
**Covariate**	***p* value**	**Odds Ratio (conf. interval 95%)**
VD supplementation (yes = 1; no = 0)	0.002	0.166 (0.053–0.517)
Use of Monoclonal Antibodies (yes = 1; no = 0)	0.007	0.227 (0.078–0.662)
Use of Mesalazine (yes = 1; no = 0)	0.046	2.968 (1.021–8.623)

## Data Availability

Data will be provided on request.
